# 1846. Quality of Life Impairment and Difficulty to Work Associated with Post-COVID Syndrome: A Cross-Sectional Study

**DOI:** 10.1093/ofid/ofad500.1674

**Published:** 2023-11-27

**Authors:** Jose J Kochuparambil, Jomel Raju

**Affiliations:** St. Joseph's College of Pharmacy, Cherthala, Pala, Kerala, India; St. Joseph's College of Pharmacy, Cherthala, Pala, Kerala, India

## Abstract

**Background:**

Following SARS-CoV-2 virus infection, patients may suffer from long-lasting symptoms regardless of disease severity. Preliminary results show limitations in health-related quality of life (HRQoL). The aim of this study is to evaluate the change in the quality of life of COVID-19-infected patients depending on the duration of the infection and the accumulation of symptoms.

**Methods:**

The study population consisted of patients (18–65 years) presenting to the Post-COVID outpatient clinic of the tertiary care hospital in India, between March and October 2022. The HRQoL was assessed by the use of the RehabNeQ and the SF-36. Data analysis was descriptive with frequencies, means, and/or percentages. In addition, a univariate analysis of variance was performed to show the dependence of physical and psychological HRQoL on specific factors. This was finally tested for significance at an alpha level of 5%.

**Results:**

Data from 318 patients were analyzed, most of whom had 3–6 months of infection (56%) and 5–10 symptoms persisted (60.4%). Both mental (MCS) and physical sum score (PCS) of HRQoL of the COVID-19 infected population were significantly lower than those of the normal population without a history of COVID-19 infection (*p* < .001). The number of remaining symptoms (MCS *p* = .0034, PCS *p* = .000) as well as the perceived ability to work (MCS p = .007, PCS p = .000), influenced the HRQoL.

**Conclusion:**

The HRQoL of patients with Post-COVID-syndrome is still reduced months after infection and so is their occupational performance. In particular, the number of symptoms could have an influence on this deficit, which would need to be further investigated. Further research is needed to detect other factors influencing HRQoL and to implement appropriate therapeutic interventions.

**Disclosures:**

**All Authors**: No reported disclosures

